# Cerebral infarct after bronchial artery embolization

**DOI:** 10.1259/bjrcr.20180087

**Published:** 2019-03-14

**Authors:** Feng Gao, Yali Xu, Shu Fang

**Affiliations:** 1 The Radiology department, tongji university affiliated shanghai First Maternity and Infant Hospital, shanghai, china; 2 The Radiology department, Shanghai Tenth People's Hospital, shanghai, china; 3 The DSA department, Shanghai Jiao Tong University School of Medicine Affiliated Renji South Hospital, Shanghai, China

## Abstract

Bronchial artery embolization is an effective and widely used procedure for controlling hemoptysis. Cerebral infarct after bronchial artery embolization is a rare and severe complication. We report two cases of cerebral infarct complicating bronchial artery embolization, most likely due to errant embolic passage through anastomoses with the ipsilateral subclavian artery.

## Introduction

Cerebral infarct after bronchial artery embolization is a rare and possibly fatal complication. We report two cases of cerebral infarct complicating bronchial artery embolization, most likely due to errant embolic passage through anastomoses with the ipsilateral subclavian artery. An approval from our Institutional Review Board for the two cases report was obtained.

## Case 1

**Figure 1 (a,b):**
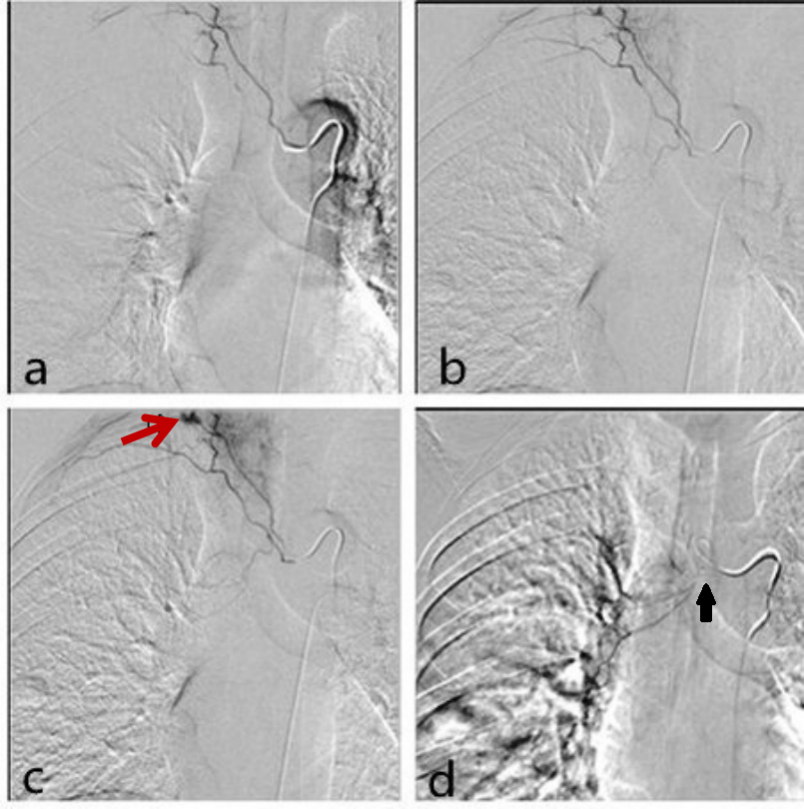
The selective bronchial angiography showed the right intercostal-bronchial artery trunk originated directly from the thoracic aorta and accompanied by the right third posterior intercostal artery and there are collateral between the third intercostal artery and the intercostal branch of the right intercostal-bronchial artery trunk, (c) an obvious anastomotic communication to subclavian artery was detected (arrow), (d) when the embolization was performed. The collateral was crossed with microcatheter (black arrow).

**Figure 2.**
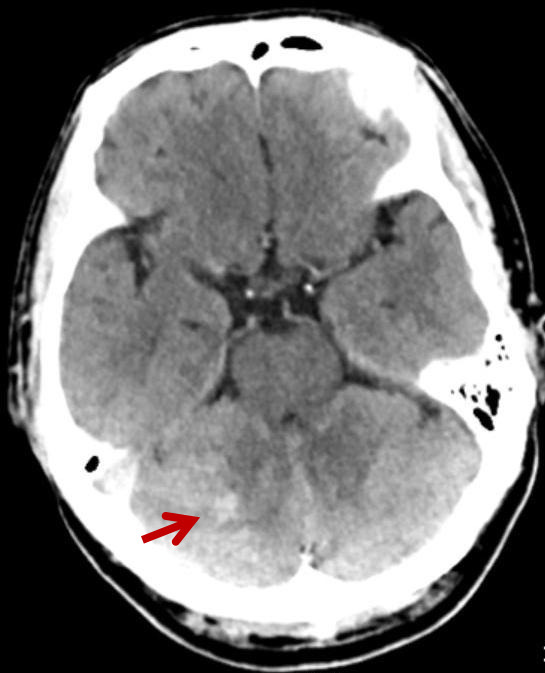
Emergency non-contrast CT showed multiple hyperdensities in the cerebellum (arrow).

**Figure 3.**
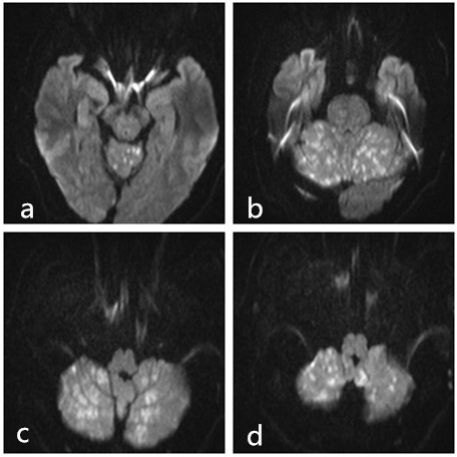
MRI showed multiple acute infarcts in the cerebellum.

59-year-old man with liver cancer presented with 2 weeks of hemoptysis of 40 ml per day. Chest CT showed metastases in the right lung. As the conservative treatment failed, the patient underwent bronchial artery embolization (BAE). The selective bronchial angiography showed the right bronchial artery had a common origin with the third right intercostal, and the dynamic images clearly demonstrated an anastomotic communication between the third intercostal artery and the right subclavian artery (Figure 1). Before the embolization was performed, the third right intercostal was crossed with microcatheter (Terumo 2.8Fr) (Figure 1-d). The right bronchial artery was embolized using lipiodol and embospheres (300–500 µm) with the patient under conscious sedation. After the embolization was performed, the patient suddenly exhibited aphasia, dyskinesia, foaming at the mouth. Emergency noncontrast CT showed multiple hyperdensities in the cerebellum (Figure 2). MRI showed multiple acute infarcts in the cerebellum (Figure 3). The patient was then transferred to the ICU, 6 hours after infarcts, the patient fell into a comatose(GCS 7) and underwent persistent high fever(persistent temperatures >°40C). Over the next 3 days, the patient developed progressive dyspnea, tachycardia. The patient died of respiratory failure 1 week later.

## Case 2

**Figure 4.**
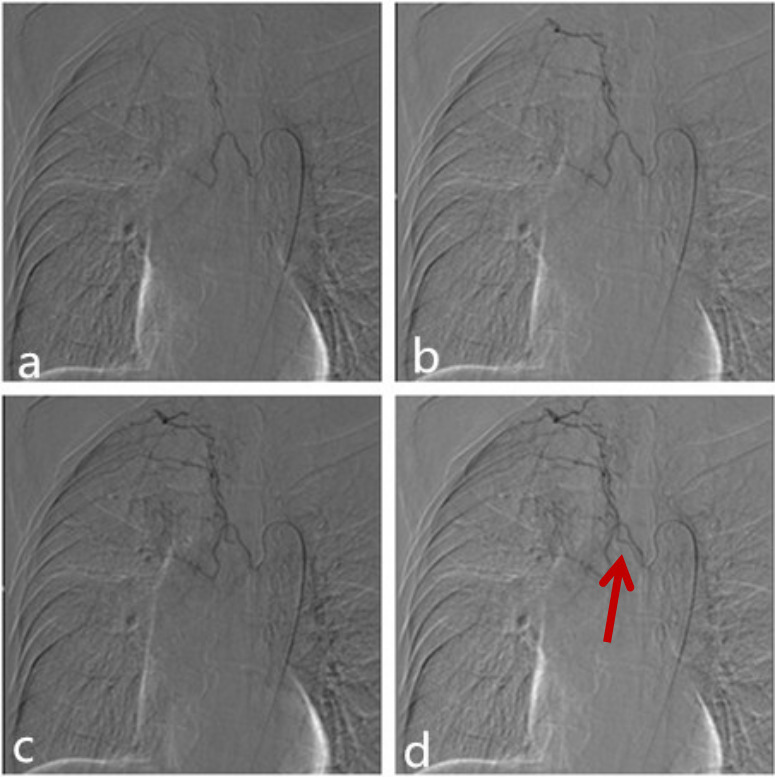
The selective bronchial angiography showed the right bronchial artery originated directly from the thoracic aorta and accompanied by the supreme intercostal artery, and there are also collateral between the supreme intercostal artery and the right bronchial artery (arrow).

**Figure 5.**
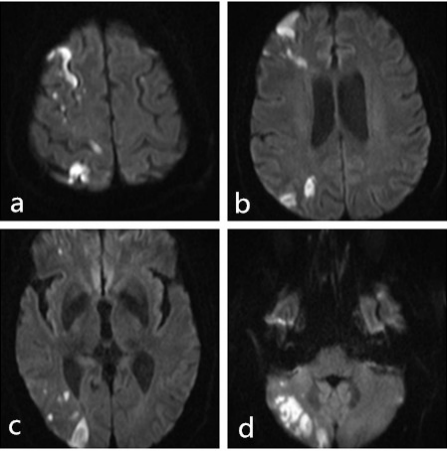
MRI showed multiple areas of infarct in the right side of the cerebrum and cerebellar hemisphere.

63-year-old male with a remote prior history of pulmonary tuberculosis presented with 10 days of persistent hemoptysis of 50 ml per day. Chest CT showed bronchiectasia in the right upper lung consistent with prior history of tuberculosis. Due to recurrent episodes of hemoptysis over the past few years, and failure of conservative treatment, the patient underwent BAE. The common bronchial trunk angiogram showed the right bronchial artery originated directly from the thoracic aorta and accompanied by the right third intercostal artery, and there are also collateral between the third intercostal artery and the right bronchial artery (Figure 4). The right bronchial artery was embolized using 300–500 µm embospheres (Merit Medical) with the patient under conscious sedation. Following recovery from the procedure, the patient exhibited somnolence, the left limb weakness (myodynamia Grade II measured by the Lovett muscle strength grading scale which Grade V represent normal). MRI showed multiple areas of infarct in the right side of the cerebrum and cerebellar hemisphere (Figure 5). The patient was treated with oxygen, dehydration of mannitol. After 3 weeks, the patient’s mental status returned to baseline and myodynamia improved to Grade III.

## Discussion

Neurologic complications due to spinal cord ischemia leading to transient or permanent paraparesis or paraplegia occurred in 0.6–4.4% of patients who performed bronchial artery embolization.^1^ Cerebral infarct after bronchial artery embolization is a rare and severe complication. It can cause serious consequences, even death. There are seven case reports published to date 2017.^2–8^ Most achieved full recovery after 2–4 weeks, however, one died of respiratory failure.^7^ As previously reported*,* the possible mechanism may be errant emboli passage through a bronchial artery–pulmonary vein shunt, or bronchial artery to vertebral artery communication, or by reflux into the vertebral artery during internal mammary or thyrocervical trunk embolization.^3,5,9–11^Jong-Ho Park reported one case of stroke after BAE due to thromboembolization in the proximal subclavian artery.^6^ Furnari described multiple anastomoses between the bronchial artery and the left subclavian artery in a patient with cystic fibrosis, but the embolization was not performed due to the high flow shunting from bronchial to left subclavian artery, and they hypothesized that pulmonary disease associated with chronic infection led to the hypertrophy of systemic vessels supplying the lungs, possibly opening multiple shunts in the pulmonary and systemic circulation.^12^

Right bronchial arteries occasionally originate directly from the aorta but more commonly share their origin with another artery, typically an intercostal artery (the third intercostal artery).^13^ In our two cases, the right bronchial arteries originated directly from the thoracic aorta and were accompanied by the third posterior intercostal artery. In Case 1, an obvious anastomosis between the right bronchial artery and subclavian was detected, and there were collaterals between the third intercostal artery and the right bronchial artery. Though the obvious anastomose between the right bronchial artery and the right subclavian artery was not detected in the other case (Case 2), collateral between the third intercostal artery and the right bronchial artery was observed. In Case 1, though the collateral was crossed when the embolization was performed, the cerebral infarction still occurred, which may have been secondary to reflux of embolic material.

**Figure 6.**
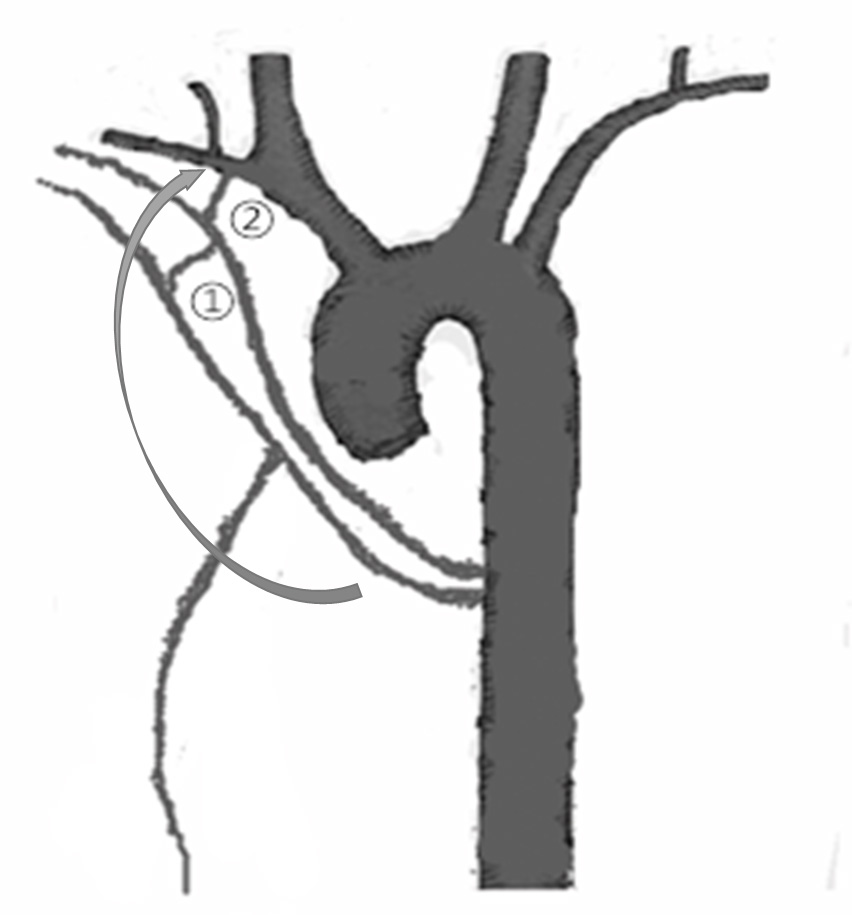
Possible mechanisms of intracranial embolization in our cases undergoing bronchial artery embolization(arrow). ①T he collateral between the third intercostal artery and the right bronchial artery. ② The anastomosis between the right bronchial artery and subclavian artery.

The mechanism of cerebral infarction in both of our cases is possibly that the embolization material entered the right subclavian artery and the right vertebral artery through the collateral when the embolization was performed, as the infarct of the Case 1 was located in the posterior circulation, and the other case’s infarct located in the right side of the cerebrum and cerebellar hemisphere (there may be laminar flow) (Figure 6). The collaterals between the bronchial artery and the subclavian artery were more likely enlarged when there is localized inflammation/infection/neoplasm. Due to the ever present risk of collaterals, arteriographic evaluation for communication with subclavian, spinal, or vertebral arteries is required when BAE is performed.

## Learning points

1. Cerebral infarct after bronchial artery embolization is a rare and possibly fatal complication.
2. Anastomotic communications may exist between the bronchial artery and intercostal arteries, that in turn, may communicate with the subclavian artery and so, embolic material may pass to the vertebral artery. Visualization of the proximal subclavian artery on a pre-embolization study should be considered a contraindication to embolization at that level.
3. Prior to BAE, the contrast study should include the most craniad extent of visualized arteries.

## Consent

Written informed consent was obtained from the patient or the patient’s family for publication of the cases report, including accompanying images.
